# Randomized Controlled Trial of Electronic Care Plan Alerts and Resource Utilization by High Frequency Emergency Department Users with Opioid Use Disorder

**DOI:** 10.5811/westjem.2015.11.28319

**Published:** 2016-01-12

**Authors:** Niels Rathlev, Reda Almomen, Ashley Deutsch, Howard Smithline, Haiping Li, Paul Visintainer

**Affiliations:** * Baystate Medical Center and Tufts University School of Medicine, Department of Emergency Medicine, Boston, Massachusetts; † ARAMCO, Department of Emergency Medicine, Dharan, Saudi Arabia; ‡ Baystate Medical Center, Department of Emergency Medicine, Springfield, Massachusetts; § Baystate Medical Center, Department of Academic Affairs Administration, Springfield, Massachusetts

## Abstract

**Introduction:**

There is a paucity of literature supporting the use of electronic alerts for patients with high frequency emergency department (ED) use. We sought to measure changes in opioid prescribing and administration practices, total charges and other resource utilization using electronic alerts to notify providers of an opioid-use care plan for high frequency ED patients.

**Methods:**

This was a randomized, non-blinded, two-group parallel design study of patients who had 1) opioid use disorder and 2) high frequency ED use. Three affiliated hospitals with identical electronic health records participated. Patients were randomized into “Care Plan” versus “Usual Care groups”. Between the years before and after randomization, we compared as primary outcomes the following: 1) opioids (morphine mg equivalents) prescribed to patients upon discharge and administered to ED and inpatients; 2) total medical charges, and the numbers of; 3) ED visits, 4) ED visits with advanced radiologic imaging (computed tomography [CT] or magnetic resonance imaging [MRI]) studies, and 5) inpatient admissions.

**Results:**

A total of 40 patients were enrolled. For ED and inpatients in the “Usual Care” group, the proportion of morphine mg equivalents received in the post-period compared with the pre-period was 15.7%, while in the “Care Plan” group the proportion received in the post-period compared with the pre-period was 4.5% (ratio=0.29, 95% CI [0.07–1.12]; p=0.07). For discharged patients in the “Usual Care” group, the proportion of morphine mg equivalents prescribed in the post-period compared with the pre-period was 25.7% while in the “Care Plan” group, the proportion prescribed in the post-period compared to the pre-period was 2.9%. The “Care Plan” group showed an 89% greater proportional change over the periods compared with the “Usual Care” group (ratio=0.11, 95% CI [0.01–0.092]; p=0.04). Care plans did not change the total charges, or, the numbers of ED visits, ED visits with CT or MRI or inpatient admissions.

**Conclusion:**

Electronic care plans were associated with an incremental decrease in opioids (in morphine mg equivalents) prescribed to patients with opioid use disorder and high frequency ED use.

## INTRODUCTION

Attendees at the 2011 Academic Emergency Medicine Consensus Conference prioritized electronic alerts and patient-specific care plans as interventions that potentially enhance the delivery of evidence-based and guideline-concordant care.^1^ The common purpose of these proposed interventions is to optimize communication between different providers working varying schedules.^2^ There is a paucity of literature supporting the use of electronic alerts for patients with high frequency emergency department (ED) use, which we define as four or more ED visits in the preceding 12 months.^3^,^4^ Based on our review of the recent peer-reviewed literature, we found only a few current, prospective studies that have examined the benefits of electronic alerts in this population.^5^,^6^

Previous studies broadly address the general population of high frequency ED users; however, given the burgeoning problem of opioid misuse and addiction in our community and across the country, we decided to focus our study on the subgroup of high frequency ED users with opioid use disorder.^7^ The latter term combines opioid abuse and dependence criteria into a single diagnosis according to the Diagnostic and Statistical Manual of Mental Disorders, Fifth Edition (DSM-5) published by the American Psychiatric Association in 2013.^8^ We believed that a randomized, controlled study to evaluate the effectiveness of electronic alert care plans was needed to demonstrate the benefits of this intervention. Our goal was to evaluate how “pushed” electronic alerts might impact the growing epidemic of prescription opioid misuse in this country.

We designed the electronic alerts in order to “push” care plan recommendations to providers as prominently visible “pop-up” screens when accessing the patients’ electronic health records. Without the electronic alerts, the provider must “pull” the information from various software applications, paper records and direct communication with primary care physicians. Accordingly, we hypothesized that use of “pushed” electronic alert care plans would help standardize care 24/7 and reduce opioid usage and other resource utilization. The primary goals were to assess the behavior of providers in terms of prescribing and administering opioids to ED and inpatients and to measure anticipated reductions in healthcare costs.

## METHODS

### Study Design

This was a randomized, non-blinded, two-group parallel design study of high frequency ED users with opioid use disorder. The researchers collecting the outcome data and the statisticians performing the analysis were blinded to the allocation. This research study received expedited approval by the investigational review board at Baystate Healthand a waiver of consent was granted since the electronic alerts presented minimal risk of harm to subjects and were implemented as standard ED practice. Our risk management department determined that signed consent for care upon presentation to the ED provided consent for participation in the electronic alerts program. Patients for whom care plans were implemented were informed of their care plans during visits to the EDs and primary care practices. The primary providers in these settings were responsible for this communication and patients could not opt out. The study was not registered at ClinicalTrials.gov since the intervention targeted provider behavior and does not involve drugs, surgical procedures, devices, behavioral treatments, educational programs, dietary interventions or quality improvement interventions.

The High Frequency User Task Force at Baystate Health System was established as a multi-disciplinary initiative to coordinate the care and create electronic alerts for patients who frequently use emergency services. The efforts of the group were primarily targeted towards individuals with opioid use disorder.^9^–^11^ The health system comprises Baystate Medical Center, an academic medical center with 114,000 annuals ED visits and two affiliated community hospitals, Mary Lane Hospital and Franklin Medical Center, with 16,000 and 27,000 annual ED visits respectively. The three institutions are located in the Pioneer Valley of western Massachusetts within a distance of no more than 40 miles from each other. The task force meets monthly and includes physicians from primary care, hospital and emergency medicine, as well as representatives from hospital-based services including case management, social services, risk management and nursing. Additional members included community partners such as Health Care for the Homeless, the Behavioral Health Network Crisis Team (community-based psychiatric outreach program) and the Springfield Coalition for Opioid Overdose Prevention (Division of the City of Springfield Department of Health and Human Services).

To minimize bias in the selection of eligible patients, we used electronic tracking of adults (age 18 and older) with four or more ED visits in the previous 12 months to the Baystate Health System to identify candidates for care plans (Figure 1–Study flow diagram). We identified candidates by review of a monthly list of patients using our Shared Medical Systems Corp. (SMS®) patient registration system, which stores patient demographic and visit data and serves as a master index for patient identification. Patients with opioid use disorder were identified by query of our SMS® patient accounting system, a hospital financial management software program that includes diagnosis, procedure and service codes for billing and collection. Patients with “Accidental poisoning by analgesics, antipyretics and antirheumatics” physician billing e-codes E850.0 (heroin), E850.1 (methadone) and E850.2 (opiates) in this accounting database were considered to meet criteria for opioid-use disorder. We used a unique patient account number to link the patient registration and accounting databases in order to create a list of eligible patients who met both criteria. In addition, potentially eligible patients with four or more ED visits in the preceding 12 months were referred to the High Frequency User Task Force by direct care providers (practicing emergency, internal or hospital medicine in our institution) who had a strong suspicion of opioid use disorder.

Patients met criteria for study based on additional supportive evidence from the following: 1) inpatient and outpatient electronic health records (Cerner FirstNet ®) including the Problem Lists e.g. for the presence of an “opiate contract” or diagnoses of “opioid dependence” or “abuse,” and, 2) the State Prescription Drug Monitoring Program. The following were criteria for exclusion: 1) significant cardiac, renal, hepatic, endocrine, metabolic, neurologic or other systemic disease (significant disease was defined as one, which, in the opinion of the principal investigator may influence the results of the study); 2) patient receiving hospice, end-of-life or comfort care only. For proposed candidates, the High Frequency User Task Force members assessed the reasons for the ED visits, reviewed the previously listed data and proposed interventions. Task force members collaborated with primary care physicians to review plans, provided input and were responsible for final determination of eligibility for the study.

Eligible patients were randomized to one of two groups (“Care Plan” group and “Usual Care” group) in a 1:1 ratio using a concealed block randomization list with a blocking factor of four. For patients randomized to the “Care Plan group,” a care plan was developed by the proposing member, reviewed by the Task Force and presented by information technology programming as an alert in the electronic health record at Baystate Health (see Appendix A for care plan template). Patients allocated to “Usual Care” group did not have a care plan or other triggers or alerts instituted. The electronic alerts appeared automatically upon initial access of the electronic health record by all ED and inpatient providers and nurses at the three affiliated hospitals.

The list of enrolled patients along with their study specific ID number, group allocation, and electronic alert implementation date were collected and managed using REDCap electronic data capture tools hosted at Tufts University.^12^ One year after enrollment, the data analyst accessed the REDCap database and collected all data for both groups before and after the intervention. We compared baseline characteristics between groups including the following: 1) age; 2) gender; 3) race; 4) presence of a primary care physician; 5) chief complaint upon presentation to the ED; 6) diagnoses documented in the past medical history; 7) presence of insurance coverage.

Between the 12-month period before and 12-month period after the implementation date, we compared changes in the following primary measures: 1) opioid medications (converted to morphine mg equivalents) prescribed to patients upon discharge, and administered to ED and inpatients. As secondary outcomes, we compared changes in: 2) total charges defined as all medical charges from all payers related to all visits to Baystate Health hospitals, 3) number of ED visits, 4) number of ED visits with advanced radiographic imaging (CT or MRI) studies; and 5) number of inpatient admissions. We included as outcomes advanced imaging and inpatient admissions since these are significant drivers of cost and typically are under the direct control of emergency providers. For example, advanced diagnostic imaging performed in the ED is reimbursed at a significant premium compared with identical outpatient exams.^13^ Moreover, hospital admission is widely considered to be the single most costly decision made by emergency providers.^14^

### Data Analysis

We conducted univariable comparisons at baseline between study groups using Fisher’s exact test for categorical variables and the Wilcoxon rank-sum test for continuous variables. To estimate change in outcomes over the two periods, a “difference-in-differences” approach was used. Repeated measures multivariable models were developed using generalized estimating equation methods with robust standard errors to account for the within-subject correlation.^15^ These models included terms for study group and period (i.e., pre vs. post), as well as a group-by-period interaction term to assess the difference in group means, across study periods. A significant p-value for this interaction term would indicate that the study means differed in their magnitude of change. We adjusted all regression models for any baseline factor that achieved a significance level of p<0.15 in the baseline comparison.

Morphine mg equivalents and total charges were log-transformed prior to multivariable analyses to account for the substantial skew in the distributions and the positive correlation between the mean and variance. Estimates for these variables were back-transformed and the ratio of their geometric means are reported with 95% confidence intervals. We analyzed and reported all other variables as a difference in adjusted means with 95% confidence intervals. The results presented are based on intention-to-treat analyses.

Sample size was estimated using the approach described by Frison and Pocock for a repeated measures analysis.^16^ A total sample size of 40 (20 patients per study group) would provide at least 80% power to detect a large effect size (Cohen’s d=0.90) for a continuous outcome (e.g., charges or morphine mg equivalents) for a two-sided test of significance at a critical level of 5%. This estimate assumes two time points (pre vs. post) and a conservative correlation among repeated measures of the outcome of 0.50. We conducted all analyses in Stata (version 13.1, StataCorp, College Station, TX).

## RESULTS

A total of 40 patients were enrolled between August, 20, 2012, and May, 29,2013. Twenty were randomized into the “Care Plan” group and 20 were randomized to the “Usual Care” group. The care plans were reviewed every six months but none required revision during the study. We excluded no eligible patients as candidates because of the following: 1) significant cardiac, renal, hepatic, endocrine, metabolic, neurologic or other systemic disease which, in the opinion of the principal investigator, would influence the results, or 2) hospice, end-of-life or comfort care only. Tables 1 and 2 list baseline patient characteristics and none of the differences were statistically significant. The only baseline covariate that met criteria for inclusion in the multivariable models was a presenting complaint of “Headache” as reported in the ED.

Table 3 shows the comparison of study groups on baseline primary and secondary measures. The results demonstrate that the groups did not demonstrate statistically significant differences at baseline as a result of the randomization process. Two individuals assigned to the “Usual Care” group inadvertently received care plans as electronic alerts. A re-analysis of the data based on “protocol-received” assignment did not alter the intention-to-treat results as presented in any meaningful way.

Table 4 shows the geometric means and proportional change for morphine mg equivalents administered to ED and inpatients, prescribed to discharged patients, as well as charges, by study group. Both study groups revealed reductions in ED and inpatient, and discharged patient opioid utilization over the study period. For ED and inpatients in the “Usual Care” group, the proportion of morphine mg equivalents received in the post-period compared with the pre-period was 15.7%, while in the “Care Plan” group the proportion received in the post-period compared with the pre-period was 4.5% (ratio=0.29, 95% CI [0.07–1.12]; p=0.07). For discharged patients in the “Usual Care” group, the proportion of morphine mg equivalents prescribed in the post-period compared with the pre-period was 25.7%. In the “Care Plan” group, the proportion prescribed in the post-period compared to the pre-period was 2.9%. The “Care Plan” group showed an 89% greater proportional change in morphine mg equivalents prescribed over the periods compared with the “Usual Care” group (ratio=0.11, 95% CI [0.01–0.092]; p=0.04).

Charges for both groups were reduced approximately 50% in the post-period compared to the pre-period, specifically 51.7% in the “Usual Care” group and 47.4% in the “Care Plan” group. Thus, the ratio of the proportional changes was 0.92; (95% CI [0.31–2.7]; p=0.88). Care plans did not alter the number of ED visits, the number of ED visits with CT or MRI studies or the number of inpatient admissions (Table 5).

## DISCUSSION

Prescription drugs and opioids specifically have taken center stage in what has become an epidemic of abuse.^17^ In 2010, an estimated seven million individuals engaged in non-medical use of prescription drugs in the United States each year.^18^ Moreover, annual deaths from prescription drug overdose have exceeded those from overdose from conventional street drugs as well as traffic accidents since 2002.^19^ To date, the efforts of policy-makers, medical providers and investigators to design and implement interventions have not been successful in reversing these trends.

With reference to the goal of studying ongoing efforts to combat opioid use disorder, our results suggest that electronic alerts prompted providers to reduce the amount of opioids prescribed to patients upon discharge from the ED and inpatient wards. In absolute terms, the incremental reduction in the “Care Plan” group was a geometric mean of −38.6 morphine mg equivalents (−85.7 versus −47.1); in context; this is equivalent to 7.8 five mg tablets of hydrocodone per patient over the course of one year.

Assuming that our results are replicable, this reduction may be significant from a clinical and public health point of view, when accounting for the multitude of patients who could benefit from electronic alerts for opioid use disorder. There were 136.3 million ED visits in the U.S. in 2011.^20^ Based on a community-based study from Oregon, authors estimated that at least 0.7% of ED visits were related to opioid use disorder in general.^2^ Specific to the non-medical use of prescription opioids, the 2011 Drug Abuse Warning Network (DAWN - Substance Abuse and Mental Health Administration) estimates that 348,000 ED visits were related to this problem.^21^ If successfully used for all of these visits, electronic alerts could-by extrapolation-conceivably eliminate the prescription of the equivalent of over 2.7 million five mg tablets of hydrocodone per year.^19^

Moreover, opioid administration decreased incrementally in absolute terms (not the geometric mean reported in Table 4) by a mean of 25.1 morphine mg equivalents (−314.8mg versus −289.7mg) with the use of electronic care plans. This change was not statistically significant; however, the analysis suggests that a larger sample size could have resulted in a statistically significant reduction in the amount of opioids administered. It should be noted that incremental reductions in both opioid administration and prescribing patterns occurred in both study groups after the implementation of electronic alerts. While this could be explained by a “carry-over” effect from the “Care Plan” group, it is more likely explained by a national trend towards decreased opioid use.

We were not able to demonstrate a statistically significant reduction in total charges or in any of the remaining measures of utilization. The difference in the geometric mean in total charges between groups was −$9,128. It should be noted that with the exception of ED visits, our outcomes point toward a favorable trend towards a reduction in resource utilization as a result of the introduction of “pushed” electronic alerts.

Mandelberg et al. discovered that, even without specific interventions, fully 62% of high frequency ED users in one year ceased to fall into that category the following year.^22^ Other investigators have likewise concluded that high frequency ED users as a group are subject to a high attrition rate from year to year.^23^ This natural pattern of high frequency ED use is patient-specific, often related to social factors and, in many cases, beyond the influence and control of our current systems of medical care. Pre-post trials are therefore subject to significant bias due to the natural ebb and flow of ED use. Accordingly, it is critical that interventions designed to manage this population are tested in a randomized controlled fashion. While high frequency ED use and interventions to address the problem have been identified as areas of research focus for many years, few rigorous comparative trials have appeared in the medical literature.^24^–^28^

Two previous studies have addressed the use of information technology to create individualized care plans for high frequency users. Both of these were pre-post trials without control groups; moreover, the trials studied a broad range of patients and were not limited to subjects at high risk of opioid use disorder. Based in hospital medicine, Mercer et al. demonstrated reductions in hospital admissions and inpatient direct costs.^5^ The second by Stokes-Buzzelli et al. was an ED trial that identified reductions in charges, laboratory tests ordered and the number of ED visits after introduction of electronic care plans.^6^ The reductions in utilization and costs demonstrated by these authors are noteworthy, but must be considered in the context of the study design. In contrast, our randomized controlled study did not find changes in total charges, ED visits or hospitalizations. The differing conclusions suggest that a randomized controlled study with a sample size larger than our current study may be required to definitively answer the questions.

## LIMITATIONS

Our study was limited to three affiliated hospitals within Baystate Health System located within a 40-mile radius of each other. We cannot exclude the possibility that patients sought care for pain-related conditions at other institutions in western Massachusetts In order to avoid “exporting” the problem to other institutions, the electronic alerts need to be adopted across a broad geographic region. The national attention on opioid use disorder and efforts by the FDA and the states to provide educational programs to limit opioid use may have impacted the numbers. In fact, both groups showed decreases in morphine mg equivalents prescribed and administered to ED and inpatients–making it more difficult to demonstrate an effect.

This should be considered to be a pilot study given the small sample size; the study has sufficient power to detect only large effects (i.e., large differences between the study groups in the change over time relative to the variability of the change) of the electronic alerts. Despite the limited power, it should be noted that, regardless of level of significance, the “Care Plan” group showed greater change in the hypothesized direction compared with the “Usual Care” group (except for number of ED visits). We suggest that a larger study will generate more precise estimates of effect.

## CONCLUSION

In an effort to combat the epidemic of opioid misuse, we implemented an intervention that was designed to influence provider prescribing practices. Our results indicate that the “pushed” electronic alerts are associated with a reduction in the dosages of morphine mg equivalent opioids prescribed to high frequency ED users patients with suspected opioid use disorder. The study did not reveal a statistically significant decrease in opioids administered during ED visits and inpatient admissions. Total charges and the numbers of total ED visits, ED visits with advanced imaging (CT or MRI) and inpatient admissions were also not reduced. “Pushed” electronic alert care plans show promise as a method of curbing the prescription of opioids, although we were unable to demonstrate an impact on other utilization measures.

## Supplementary Information



## Figures and Tables

**Figure:**
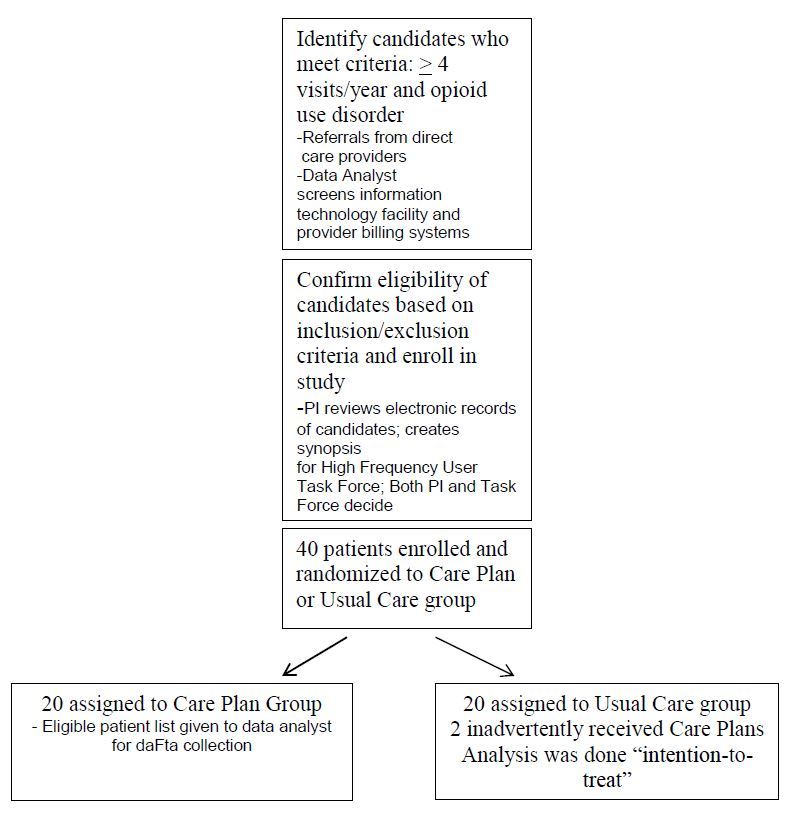
Study flow diagram. *PI,* principal investigator

**Table 1:** Demographic characteristics of study groups.

	Usual care group (N=20)		Care plan group (N=20)		
		
Baseline characteristics	%	(n)	%	(n)	p
Male	35.0	(7)	60.0	(12)	0.21
Race					
Caucasian	50.0	(10)	60.0	(12)	0.83
African-American	30.0	(6)	25.0	(5)	
Hispanic	20.0	(4)	15.0	(3)	
Age, mean (sd)	47.9	(11.5)	44.7	(10.5)	0.37

**Table 2:** Baseline clinical characteristics of study groups.

	No care plan (N=20)		Care plan (N=20)		
		
Baseline characteristics	%	(n)	%	(n)	P
Primary care physician	95.0	(19)	95.0	(19)	1.0
Chief complaint					
Back pain	60.0	(12)	45.0	(9)	0.53
Headache	50.0	(10)	20.0	(4)	0.10
Abdominal pain	95.0	(19)	85.0	(17)	0.60
Chest pain	55.0	(11)	45.0	(9)	0.75
Chronic medical condition					
IV drug use	10.0	(2)	25.0	(5)	0.41
Diabetes	30.0	(6)	50.0	(10)	0.33
Renal failure	10.0	(2)	10.0	(2)	1.00
Coronary artery disease	20.0	(4)	15.0	(3)	1.00
Gastrointestinal disorder	45.0	(9)	65.0	(13)	0.34
HIV	0.0	(0)	5.0	(1)	1.00
Cocaine	20.0	(4)	20.0	(4)	1.00
Alcohol	30.0	(6)	35.0	(7)	1.00
Anxiety	75.0	(15)	65.0	(13)	0.73
Depression	70.0	(14)	85.0	(17)	0.45
PTSD	25.0	(5)	20.0	(4)	1.00
Psychosis	25.0	(5)	50.0	(10)	0.19
Insurance coverage	50.0	(10)	45	(9)	1.00

*IV,* intravenous; *HIV,* human immunodeficiency virus; *PTSD,* post-traumatic stress disorder

**Table 3:** Baseline comparison of primary and secondary measures per patient for the year prior to study entry.

	Usual care group (N=20)		Care plan group (N=20)		
		
Utilization measure per patient	*median*	*(range)*	*median*	*(range)*	P
Morphine mg equivalents					
Administered to ED/inpatients	540.7	(27.5, 1529.3)	551.2	(0.0, 5008.3)	0.45
Prescribed to discharged patients	100.0	(0.0, 757.5)	285.0	(0.0, 976.5)	0.32
Charges	$34,905	($0, $191,174)	$42,035	($2,200, $250,184)	0.59
Number of ED visits	17.5	(4, 50)	20.5	(4, 62)	0.68
Number of ED visits with CT/MRIs	5	(0, 31)	8.5	(1, 51)	0.61
Number of admissions	2	(0, 22)	2	(0, 17)	0.35

*ED,* emergency department; *CT,* computed tomograph; *MRI,* magnetic resonance imaging

**Table 4:** Proportional change in selected outcomes per patient by study group.

	Usual care group			Care plan group				
		
Utilization measure per patient	Pre	Post	Post/pre (%)	Pre	Post	Post/pre (%)	Ratio (95%CI)	p
Morphine mg equivalents								
Administered to ED/inpatients	343.7	54.0	15.7	329.8	15.0	4.5	0.29 (0.07, 1.12)	0.07
Prescribed to discharged patients	63.4	16.3	25.7	88.2	2.5	2.9	0.11 (0.01, 0.92)	0.04
Charges ($)	27,465	14,201	51.7	42,605	20,213	47.4	0.92 (0.31, 2.7)	0.88

*ED,* emergency department

**Table 5:** Comparison of change in utilization measures per patient over time by study group.

	Usual care group (N=20)		Care plan group (N=20)		
		
Utilization measure per patient	*mean*	*(95% CI)*	*mean*	*(95% CI)*	P
Number of ED visits	−12.8	(−19.8, −5.8)	−10.7	(−17.5, −4.0)	0.68
Number of ED visits with CT/MRI	−5.8	(−9.1, −2.5)	−5.7	(−10.0, −1.4)	0.98
Number of admissions	−1.3	(−2.8, 0.2)	−2.6	(−5.0, −0.2)	0.46

*ED,* emergency department; *CT,* computed tomography; *MRI,* magnetic resonance imaging

## References

[1] Kocher K, Shane S, Venkatesh AK (2011). Interventions to safeguard effectiveness during periods of emergency department crowding. Acad Emerg Med.

[2] Zechnich AD, Hedges JR (1996). Community-wide emergency department visits by patients suspected of drug-seeking behavior. Acad Emerg Med.

[3] Locker TE, Boston S, Mason SM (2007). Defining frequent use of an urban emergency department. Emerg Med J.

[4] Weber EJ (2012). Defining frequent use: The numbers no longer count. Ann Emerg Med.

[5] Mercer T, Bee J, Velazquez M (2015). The highest utilizers of care: Individualized care plans to coordinate care, improve healthcare service utilization, and reduce costs at an academic tertiary care center. J Hosp Med.

[6] Stokes-Buzzelli S, Peltzer-Jones JM, Martin GB (2010). Use of health information technology to manage frequently presenting emergency department patients. West J Emerg Med.

[7] Okie S (2010). A flood of opioids, a rising tide of deaths. N Engl J Med.

[8] American Psychiatric Association (2013). Diagnostic and Statistical Manual of Mental Disorders, Fifth Edition (DSM-5). Highlights of changes from DSM-IV-TR to DSM-5.

[9] Dunford JV, Castillo EM, Chan TC (2006). Impact of the San Diego serial inebriate program on use of emergency medical resources. Ann Emerg Med.

[10] Duong D, Rathlev NK, McGrath M (2012). Does mandatory inpatient alcohol detoxification reduce emergency department recidivism, hospital admissions and emergency medical service transports for patients with chronic, severe alcohol dependence?. J Emerg Med.

[11] Hunt KA, Weber EJ, Showstack JA (2006). Characteristics of frequent users of emergency departments. Ann Emerg Med.

[12] Harris PA, Taylor R, Thielke R (2009). Research electronic data capture (REDCap) - A metadata-driven methodology and workflow process for providing translational research informatics support. J Biomed Inform.

[13] Wynn BO Medicare payment for hospital outpatient services: A historical review of policy options.

[14] Schuur JD, Baugh CW, Hess EP (2011). Critical pathways for post-emergency outpatient diagnosis and treatment: Tools to improve the value of emergency care. Acad Emerg Med.

[15] Singer JD, Willett JB (2003). Applied Longitudinal Data Analysis.

[16] Frison L, Pocock SJ (1992). Repeated measures in clinical trials: Analysis using mean summary statistics and its implications for design. Stat Med.

[17] McDonald DC, Carlson KE (2013). Estimating the Prevalence of Opioid Diversion by “Doctor Shoppers” in the United States. PLoS One.

[18] Substance Abuse and Mental Health Services Administration (2011). Results from the 2010 National Survey on Drug Use and Health: Summary of National Findings.

[19] Paulozzi LJ, Budnitz DS, Xi Y (2006). Increasing deaths from opioid analgesics in the United States. Pharmacoepidemiol Drug Saf.

[20] Emergency Department Visits Centers for Disease Control and Prevention Website.

[21] Substance Abuse and Mental Health Services Administration, Drug Abuse Warning Network (2013). 2011: National Estimates of Drug-Related Emergency Department Visits.

[22] Mandelberg JH, Kuhn RE, Kohn MA (2000). Epidemiologic analysis of an urban, public emergency department’s frequent users. Acad Emerg Med.

[23] Kne T, Young R, Spillane L (1998). Frequent ED users: patterns of use over time. Am J Emerg Med.

[24] Spillane LL, Lumb EW, Cobaugh DJ (1997). Frequent users of the emergency department: Can we intervene?. Acad Emerg Med.

[25] Lucas RH, Sanford SM (1998). An analysis of frequent users of emergency care at an urban university hospital. Ann Emerg Med.

[26] Pope D, Fernandes CMB, Bouthillette (2000). Frequent users of the emergency department: a program to improve care and reduce visits. CMAJ.

[27] Althaus F, Paroz S, Hugli O (2011). Effectiveness of interventions targeting frequent users of emergency departments: A systematic review. Ann Emerg Med.

[28] Raven MC (2011). What we don’t know won’t hurt us: Interventions for frequent emergency department users. Ann Emerg Med.

