# Supplementary material for: Randomized Controlled Trial of Electronic Care Plan Alerts and Resource Utilization by High Frequency Emergency Department Users with Opioid Use Disorder
Source: West J Emerg Med. 2016 Jan 12;17(1):28–34. doi: 10.5811/westjem.2015.11.28319 (PMC4729415; doi:10.5811/westjem.2015.11.28319)
Supplement: Supplementary file 1 [file wjem-17-28-s001.pdf]

**Appendix A: Care Plan Template**

**Care Plan For:** ____________________

**Medical Record Number:** ____________________

**Date of Birth:** ____________________

## Rationale:

------- has presented to the ED on x occasions in the past 12 months for a variety of complaints related to -----. (S)he has received x prescriptions for opioids in the past year.

(S)he has a history of -----------. (S)he is currently being treated with x.

The patient states that (s)he is allergic to .

## Interventions:

The patient will be treated with opiates only for serious acute medical disorders, but not for other presentations. The patient will not receive prescriptions for oral opiate medications for chronic pain from the ED.

## Goals and Expected Outcomes:

The goal is to refer the patient for follow-up with a primary care physician. Her(his) care plan will be reviewed every 6 months.

| | | |
|---|---|---|
| **Submitted by:** | | |
| **Approved by Task Force:** | ☐ **Yes** ☐ **No** | **Effective Date:** |
| **PCP/Group:** | **Name: None**<br>**Phone #** | **Discussed with PCP?**<br>☐ **Yes** ☐ **No** |
| **Discussed/Mailed w/Patient.** | ☐ **Discussed** ☐ **Mailed** | **Date:** |
| **Due for Review:** | | |
